# Preclinical evaluation of *(S)*-[^18^F]GE387, a novel 18-kDa translocator protein (TSPO) PET radioligand with low binding sensitivity to human polymorphism rs6971

**DOI:** 10.1007/s00259-021-05495-w

**Published:** 2021-08-18

**Authors:** Nisha K. Ramakrishnan, Matthew Hird, Stephen Thompson, David J. Williamson, Luxi Qiao, David R. Owen, Allen F. Brooks, Peter J. H. Scott, Sergio Bacallado, John T. O’Brien, Franklin I. Aigbirhio

**Affiliations:** 1 Molecular Imaging Chemistry Laboratory, Wolfson Brain Imaging Centre, Department of Clinical Neurosciences, University of Cambridge, Biomedical Campus, Cambridge, CB2 0SZ UK; 2 Department of Brain Sciences, Imperial College London, Hammersmith Hospital, London, UK; 3 Division of Nuclear Medicine, Department of Radiology, University of Michigan Medical School, 1301 Catherine Street, Ann Arbor, MI 48109 USA; 4 Statistical Laboratory, Centre for the Mathematical Sciences, University of Cambridge, Wilberforce Rd., Cambridge, CB3 0WB UK; 5 Department of Psychiatry, School of Clinical Medicine, University of Cambridge, Cambridge Biomedical Campus, Cambridge, UK

**Keywords:** Neuroinflammation, TSPO, Polymorphism, Positron emission tomography, Brain, GE387

## Abstract

**Purpose:**

Positron emission tomography (PET) studies with radioligands for 18-kDa translocator protein (TSPO) have been instrumental in increasing our understanding of the complex role neuroinflammation plays in disorders affecting the brain. However, (*R*)-[^11^C]PK11195, the first and most widely used TSPO radioligand has limitations, while the next-generation TSPO radioligands have suffered from high interindividual variability in binding due to a genetic polymorphism in the TSPO gene (rs6971). Herein, we present the biological evaluation of the two enantiomers of [^18^F]GE387, which we have previously shown to have low sensitivity to this polymorphism.

**Methods:**

Dynamic PET scans were conducted in male Wistar rats and female rhesus macaques to investigate the in vivo behaviour of (*S*)-[^18^F]GE387 and (*R*)-[^18^F]GE387. The specific binding of (*S*)-[^18^F]GE387 to TSPO was investigated by pre-treatment with (*R*)-PK11195. (*S*)-[^18^F]GE387 was further evaluated in a rat model of lipopolysaccharide (LPS)-induced neuroinflammation. Sensitivity to polymorphism of (*S*)-GE387 was evaluated in genotyped human brain tissue.

**Results:**

(*S*)-[^18^F]GE387 and (*R*)-[^18^F]GE387 entered the brain in both rats and rhesus macaques. (*R*)-PK11195 blocked the uptake of (*S*)-[^18^F]GE387 in healthy olfactory bulb and peripheral tissues constitutively expressing TSPO. A 2.7-fold higher uptake of (*S*)-[^18^F]GE387 was found in the inflamed striatum of LPS-treated rodents. In genotyped human brain tissue, (*S*)-GE387 was shown to bind similarly in low affinity binders (LABs) and high affinity binders (HABs) with a LAB to HAB ratio of 1.8.

**Conclusion:**

We established that (*S*)-[^18^F]GE387 has favourable kinetics in healthy rats and non-human primates and that it can distinguish inflamed from normal brain regions in the LPS model of neuroinflammation. Crucially, we have reconfirmed its low sensitivity to the TSPO polymorphism on genotyped human brain tissue. Based on these factors, we conclude that (*S*)-[^18^F]GE387 warrants further evaluation with studies on human subjects to assess its suitability as a TSPO PET radioligand for assessing neuroinflammation.

**Supplementary Information:**

The online version contains supplementary material available at 10.1007/s00259-021-05495-w.

## Background

Neuroinflammation is a potential contributing factor to several disorders affecting the brain. Whether it is sudden in onset like traumatic brain injury, intermittent like multiple sclerosis or progressive as with neurodegenerative disorders, post-mortem studies have shown the presence of markers of inflammation [1–3]. In progressive disorders such as Alzheimer’s disease or Parkinson’s disease, it has not been established whether neuroinflammation plays a causative or reactive role in the neurodegenerative process. Prospective imaging studies of neuroinflammation in patients are critical to addressing these questions and going forward, such studies are expected to increase our understanding of the role that neuroinflammation plays in these complex diseases.

Positron emission tomography (PET) studies using radioligands for neuroinflammation have begun to highlight the key role of neuroinflammation in brain disorders [4] (for a recent review of 18-kDa translocator protein (TSPO) PET, see [5]). TSPO is present on the outer mitochondrial membrane of microglia and is upregulated in rodents when microglia are activated in the presence of neuroinflammatory stimuli [6]. The first and most widely studied of the TSPO PET radioligands is (*R*)-[^11^C]PK11195, which has been extensively used in preclinical and clinical studies. However, it suffers from limitations, including poor signal-to-noise ratio, difficult radiosynthesis and a short 20-min half-life due to being radiolabelled with carbon-11 [7]. As a result, second-generation radioligands have been developed to address these issues. However, most of these second-generation radioligands have suffered from a high interindividual variability in binding due to sensitivity to the rs6971 polymorphism of the TSPO gene [8]. This polymorphism results in high affinity binders (HABs), low affinity binders (LABs) and mixed affinity binders (MABs) towards these radioligands in the general human population, with LAB to HAB binding ratios that vary from 55.3 to 4.0 (see Table 1), while the first-generation radioligand [^11^C]PK11195 has a low binding ratio of approximately 1 [9]. This variation of binding affinities can complicate quantification of the acquired PET data, requiring all study participants to be genotyped and LABs (and potentially even MABs) to be excluded from clinical imaging studies. As well as complicating study design and logistics, this both limits the wider generalisability of findings generated from imaging studies, which only include HABs, and also hinders potential future translation to clinical practice.

**Table 1:** LAB to HAB binding ratios for major TSPO PET radioligands and more recent radioligands at the preclinical evaluation stage

Radioligand	LAB/HAB ratio	Reference
Clinically tested radioligands		
Short half-life carbon-11 labelled		
[^11^C]PK11195	0.8	[9]
[^11^C]PBR28	55.3	[9]
[^11^C]DPA-713	4.4	[9]
[^11^C]ER176	1.3	[10]
Longer half-life fluorine-18 labelled		
[^18^F]PBR06	17.3	[9]
[^18^F]PBR111	4.0	[9]
[^18^F]GE180	15.2	[11, 12]
[^18^F]DPA714	No publications	
Radioligands at preclinical stage		
(*R*)-[^18^F]NEBIFQUINIDE	1.1	[13]
[^18^F]LW223	1	[14]

As a result, with the increasing interest in longitudinal imaging studies to elucidate the role of neuroinflammation in various disorders of the brain, there is an urgent need to develop TSPO radioligands which have very low variability of binding affinity stemming from this TSPO polymorphism. In addition, fluorine-18-labelled radioligands are required to enable simpler productions that are more efficient and provide wider availability.

Towards this objective, we have identified a novel TSPO ligand, GE387, which has been shown to have a low LAB to HAB binding ratio (1.3) and have developed a method for its radiolabelling with fluorine-18, including chemical and radiochemical analysis [15]. Herein, further biological evaluation of the two enantiomers of [^18^F]GE387 is presented. We explored the in vivo behaviour of (*S*)-[^18^F]GE387 and (*R*)-[^18^F]GE387 in healthy male rats and healthy female rhesus macaques and assessed the (*S*)-[^18^F]GE387 in a rat model of neuroinflammation induced by unilateral injection of LPS into the striatum and further evaluated its LAB to HAB binding ratio in genotyped human brain tissue.

## Materials and methods

### Radiochemistry

(*S*)-[^18^F]GE387 and (*R*)-[^18^F]GE387 were synthesised from their corresponding enantiomerically pure precursors using the method previously reported (see supplementary information) [15].

### Animals

#### Rats

Male Wistar rats (Charles River Laboratories, UK) were housed in Techniplast 2000P IVC cages on a layer of Aspen bedding in a room with constant temperature (21 ± 2 °C) and fixed 12 h light–dark regime (lights on at 7:00 am). Food and water were available ad libitum. After arrival, the rats were allowed to acclimatise for at least 7 days before any procedures were performed (details of the number of rats used in Supplementary Table 1). This research was regulated under the Animals (Scientific Procedures) Act 1986 Amendment Regulations 2012 following ethical review by the University of Cambridge Animal Welfare and Ethical Review Body (AWERB).

#### Monkeys

The University of Michigan PET Center has maintained 2 rhesus macaques for ~ 17 years and the monkeys are individually housed in adjacent steel cages (83.3 cm high × 152.4 cm wide × 78.8 cm deep) equipped with foraging boxes. They are currently housed in adjacent cages as repeated attempts to socially house them in the same cage have been unsuccessful due to aggressive incompatibility. Cages are metal and do contain gridded floors for radiation safety reasons (radioactive waste is contained to the gridded floor and is easier to clean). Temperature and humidity are carefully controlled, and the monkeys are kept on a 12 h light/12 h dark schedule. Monkeys are fed Lab Fiber Plus Monkey Diet (PMI Nutrition Intl. LLC, Shoreview, MN, USA) that is supplemented with fresh fruit and vegetables daily. Water and enrichment toys (manipulanda and food-based treats) are available continuously in the home cage.

### PET scans in rats

The animals were anaesthetised using isoflurane at a concentration of 5% in O_2_, and anaesthesia was maintained using 1.5–2.5% isoflurane in O_2_. The femoral vein and artery were cannulated for radioligand injection and arterial blood sampling respectively. An incision was made in the skin parallel to the femoral artery. The femoral artery was separated from the femoral vein and both were temporarily ligated to prevent leakage of blood. A small incision was made in the vein, and a cannula (0.8 mm outer diameter and 0.4 mm inner diameter) attached to a syringe filled with heparinised saline was placed into it. The cannula was checked for patency and secured to the vein with a suture. The artery was similarly cannulated and the wound closed. For blocking experiments, (*R*)-PK11195 (1 mg/kg) was injected slowly via the femoral vein 30 min before the scan started. The anaesthetised rat was placed on the bed of a microPET Focus 120 scanner (modified in-house) [16]. The rat was positioned in the transaxial position with its head in the field of view on a heating mat and the heart rate monitored. A transmission scan with a ^68^Ge point source (515 s) was performed for attenuation and scatter correction of 511 keV photons. The radioligand, formulated in saline, was injected via the femoral vein cannula and the emission scan started simultaneously. A list-mode protocol was used with a total acquisition time of 60 min.

Seventeen arterial blood samples (50–150 μL) were collected at approximately 5, 10, 15, 30, 45, 60, 75, 90 and 105 s and 2, 3, 5, 7.5, 10, 15, 30 and 60 min post-injection and these samples immediately placed on ice. A sample of whole blood (25 μL) was reserved, and the remainder centrifuged (Eppendorf 5430-R centrifuge, at 5 °C, 5 min, 30,130 RCF) to obtain plasma. Whole blood and plasma radioactivity were measured using a gamma counter (Hidex AMG 425–601).

Plasma samples (3, 5, 10, 15, 30 and 60 min) and a hemisphere of the brain (60 min) dissected out during the biodistribution (see below) and placed on the ice were further processed for metabolite analysis. An equal volume of ice-cold acetonitrile was added to the samples and the plasma mixture was vortexed (5–10 s) while the brain mixture was homogenised using a bead mill (Fisher Scientific; Bead Mill 4) and then centrifuged (5 °C, 5 min, 30,130 RCF). The supernatant was directly injected through a 200-μL loop into a Thermo Scientific Ultimate 3000 HPLC system with a Capcell PAK, UG120, 5 μm, 4.6 × 250 mm column. A linear gradient of acetonitrile:water from 10 to 80%, at a flow rate of 1.5 mL min^−1^ over 14 min was used. The eluate was passed through a Berthold Flowstar LB 513 radiodetector and fractions collected from the outlet every minute and radioactivity present in the samples was measured using a gamma counter. The parent radioligand concentration was expressed as a percentage of the total plasma radioactivity.

At the end of the scan, the animals were culled while under deep anaesthesia and blood, various brain regions and peripheral tissues were dissected out for biodistribution study. The radioactivity in the samples was measured and the samples weighed using a gamma counter. The results were expressed as dimensionless standardised uptake values (SUV = [(tissue activity) × (body weight)]/injected dose). SUVs were calculated assuming a specific gravity of 1 g mL^−1^ for brain tissue. All results are expressed as mean ± SEM. Differences between groups were examined separately for brain regions and peripheral tissues using 2-way ANOVA followed by a post hoc Bonferroni test.

Image reconstructions were performed using microPET Manager 2.4.1.1, ASIPro 6.7.1.2 (Siemens). Acquisition data were then Fourier re-binned in 22 time frames (6 × 10 s, 4 × 30 s, 4 × 60 s, 1 × 180 s, 4 × 300 s, 3 × 600 s) and the data were reconstructed per time frame employing an iterative reconstruction algorithm (ordered subsets expectation maximisation, OSEM 2D with Fourier re-binning, four iterations, and 16 subsets). The final datasets consisted of 95 slices with a slice thickness of 0.8 mm and an in-plane image matrix of 128 × 128 pixels. Voxel size was 0.8 × 0.8 × 0.8 mm. Datasets were corrected for decay, random coincidences, scatter and attenuation.

Three-dimensional volumes-of-interest (VOIs) for rat brain MRI template available on PMOD software (version 3.8; PMOD technologies, Zurich, Switzerland) were modified to obtain VOIs for the whole brain and smaller brain regions. Individual PET images were then co-registered with this MRI template and the VOI transferred from MRI to PET. Brain time–activity curves (TACs) were obtained for each of the animals and the results expressed as SUV.

### LPS neuroinflammation model

Male Wistar rats were anaesthetised using isoflurane at a concentration of 5% in O_2_, and anaesthesia was maintained using 1.5–2.5% isoflurane in O_2_. Animals were prepared for sterile surgery and administered analgesic buprenorphine (Temgesic®). The rats were placed on a stereotactic frame (Kopf) and an incision was made to expose the bregma. The skull was drilled bilaterally (Bregma + 0.7 mm anterior and ± 3.0 mm lateral) and lipopolysaccharide (LPS) (in saline 2.5 μg/μL, 4 μL) was injected (− 5.5 mm from the surface of the brain) into one striatum and saline into the contralateral striatum using a Hamilton syringe at the rate of 0.5 μL min^−1^. After waiting 2 min at the end of the injection, the needle was slowly withdrawn and the incision closed, and the animals recovered from anaesthesia and placed in cages with soft surgical bedding. Mash food was made available and analgesic buprenorphine was administered subcutaneously b.i.d. as necessary. PET scans were performed, as described previously, 3 days after the LPS injection. VOIs were drawn on the left and right striatum on the MRI template and TACs obtained as above.

### Kinetic modelling of rat PET data

Two-tissue compartment model (2-TCM) was fitted to the dynamic PET data using PMOD software (version 3.8; PMOD technologies, Zurich, Switzerland). Blood and plasma TACs were interpolated by fitting a 3-exponentials model. Where whole blood or plasma curves were not available (due to metabolite sampling protocol or failure of the cannula), population averages for the group corrected for injected dose and weight of the individual animal were used. Group population average metabolite curve fractions were used for correcting plasma curves. The parameter for cerebral blood volume was fixed at 3.6% [17] and blood delay was fitted and the rate constants *K*_1_, *k*_2_, *k*_3_ and *k*_4_ were estimated from the curve fit. Macro parameters, non-displaceable volume of distribution (*V*_ND_) was calculated as *K*_1_/*k*_2_, total volume of distribution (*V*_T_) as *K*_1_/*k*_2_ × (1 + *k*_3_/*k*_4_) and non-displaceable binding potential (BP_ND_) as *k*_3_/*k*_4_.

The effect of pre-treatment with (*R*)-PK11195 on the kinetics of (*S*)-[^18^F]GE387 was investigated in the whole brain and in the olfactory bulb. The effect of LPS-induced acute neuroinflammation was investigated by comparing the kinetic modelling parameters obtained from the ipsilateral and contralateral striatum. The contralateral striatum was also compared with the striatum of control animals. Difference between groups for each of the parameters was analysed using 2-way ANOVA followed by Bonferroni post hoc analysis.

### PET scans in non-human primates

Non-human primate studies were performed in accordance with the standards set by the Institutional Animal Care and Use Committee (IACUC) at the University of Michigan.

Imaging was done on two mature female rhesus macaques (*n* = 2, weight = 8.81 ± 0.64 kg). Each animal was scanned with both (*S*)-[^18^F]GE387 and (*R*)-[^18^F]GE387 on separate days. The animal was anaesthetised in the home cage with ketamine (5–20 mg/kg *i.m.*) and transported to the PET imaging suite. The monkey was intubated for mechanical ventilation, and anaesthesia was continued with isoflurane (1–5% in oxygen). Anaesthesia was maintained throughout the duration of the PET scan. A venous catheter was inserted into one hind limb, and the primate was placed on the PET gantry of a Concorde MicroPET P4 scanner with the head located in the centre of the field of view and secured to prevent motion artefacts. Following a transmission scan (cobalt-57 rod source), each animal was administered either (*S*)-[^18^F]GE387 or (*R*)-[^18^F]GE387 as an intravenous bolus over 1 min and imaged for 120 min using Michigan’s standard fluorine-18 imaging protocol (5 × 1 min frames; 2 × 2.5 min frames; 2 × 5 min frames; 10 × 10 min frames). The final datasets consisted of 63 slices with a slice thickness of 1.2 mm and an in-plane image matrix of 128 × 128 pixels. Pixel size was 1.4 × 1.4 mm.

Emission data were corrected for attenuation and scatter and reconstructed using the 3D maximum a priori (3D MAP) method. Since there was no MRI or CT data available for these animals, the National Institute of Mental Health Macaque Template [18] was used as a guide to determine regions of interest (ROIs) for the whole brain, striatum, thalamus, cortex, and cerebellum. Previous PET datasets (e.g. [^11^C]flumazenil) in the same primates were used as a secondary anatomical reference to confirm placing of ROIs. The (*S*)-[^18^F]GE387 and (*R*)-[^18^F]GE387 images were co-registered to the [^11^C]flumazenil scans for confirming volumetric ROIs. Finally, by using a summed image, ROIs were drawn on multiple planes, and the volumetric ROIs were then applied to the full dynamic dataset to generate time–radioactivity curves. Whole-brain TACs were obtained for each of the animals. The results were expressed as SUV, as described above.

### Competition binding assay

The competition binding assay using genotyped human cortical brain tissue obtained from UK MS brain bank, as described by Owen et al. [8], was used to corroborate our previous data obtained in human embryonic kidney cell lines [15] showing that GE387 has low sensitivity to the rs6971 TSPO polymorphism. Briefly, aliquots of membrane suspension obtained from individual genotyped human brain tissue from both high affinity binders (HABs) and low affinity binders (LABs) (*n* = 7 each) was incubated with [^3^H]PK11195 (2 nM) and unlabelled (*S*)-GE387 (10 concentrations ranging from 0.1 nM to 3 μM, in triplicate) at room temperature for 60 min. At the end of the incubation period, the suspension was filtered and washed, and the individual filter papers placed in vials before adding scintillation fluid (Perkin Elmer Ultima Gold MV) and counting in a Perkin Elmer MicroBeta Trilux liquid scintillation counter after a minimum of 30 min of being in darkness. The competition binding data was analysed using the iterative nonlinear regression curve-fitting software supplied with GraphPad Prism 5.0. The log concentrations of (*S*)-GE387 were plotted against counts from the individual HABs and LABs to obtain IC_50_ values. *K*_*i*_ values for (*S*)-GE387 were calculated from IC_50_ values according to the Cheng and Prusoff equation [19], using a dissociation constant (*K*_*d*_) of 29.25 nM for ^3^H-PK11195 [8]. The *K*_*i*_ values were in turn used to calculate LAB to HAB ratio.

## Results

### PET scans in rats

**Fig. 1:**
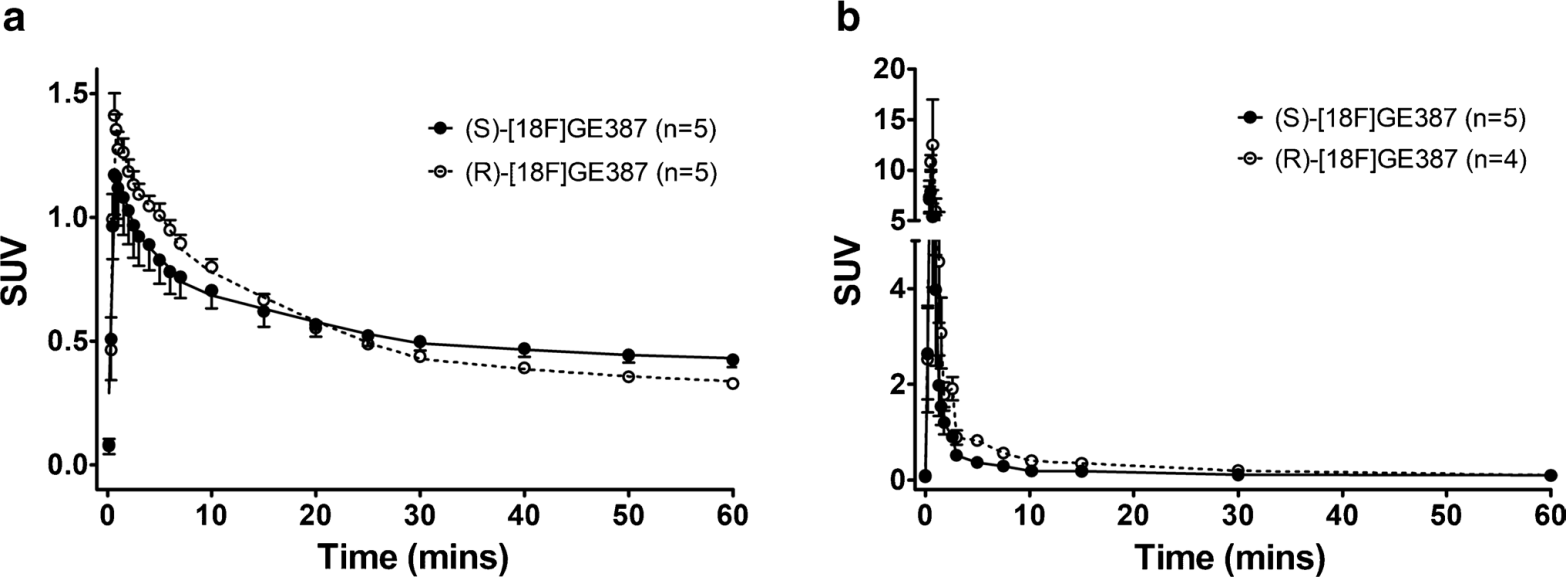
**a** Brain and **b** metabolite-corrected plasma time–activity curves of (*S*)-[^18^F]GE387 and (*R*)-[^18^F]GE387 in naïve healthy rats. Both radioligands entered the brain with a peak within the first minute and the *R*-enantiomer had a comparatively faster washout. (*R*)-[^18^F]GE387 had higher plasma exposure than (*S*)-[^18^F]GE387

PET scan images of the brains of the healthy rats showed that the uptake of both (*R*)-[^18^F]GE387 and (*S*)-[^18^F]GE387 was low as expected in healthy naïve rats, with the olfactory bulb showing the highest uptake. Whole-brain time–activity curves (TACs) for the rats scanned with (*S*)-[^18^F]GE387 and (*R*)-[^18^F]GE387 revealed rapid brain uptake (Fig. 1a) with a peak SUV of 1.1 for (*S*)-[^18^F]GE387 and 1.4 for (*R*)-[^18^F]GE387. Maximal brain uptake was within 1 min for both radioligands followed by a slightly faster washout for the *R*-enantiomer (AUC = 32.7 ± 1.4) than the *S*-enantiomer (AUC = 33.6 ± 2.9), but this difference was not found to be statistically significant. The brain TACs were similar to those obtained for another TSPO radioligand, [^18^F]DPA714, in our laboratory (supplementary information).

The metabolite-corrected plasma curves of both (*S*)-[^18^F]GE387 and (*R*)-[^18^F]GE387 (Fig. 1b) showed rapid clearance of both radioligands from the blood pool. The plasma curves were bi-exponential in nature with a peak around 30–45 s post-injection. The plasma exposure of (*R*)-[^18^F]GE387 (AUC = 27.52 ± 3.9) was statistically higher (*P* = 0.0385) than that for (*S*)-[^18^F]GE387 (AUC = 16.30 ± 2.4).

Plasma metabolite analysis showed that both enantiomers were metabolised with the time-dependent formation of at least two radiometabolites. These polar radiometabolites eluted at shorter retention times than the parent radioligand. (*R*)-[^18^F]GE387 was slightly less stable in plasma with 20% parent remaining at 60 min, compared to 28% for (*S*)-[^18^F]GE387. Brain metabolite analysis following a single extraction indicated the presence of a polar metabolite in the brain, with this metabolite being present at significantly higher amounts for the (*R)-*[^18^F]GE387-enantiomer. In rats scanned with (*R*)-[^18^F]GE387, 47% intact parent was found in the brain, compared to 78% intact parent in the brain for (*S*)-[^18^F]GE387 at 60 min.

**Fig. 2:**
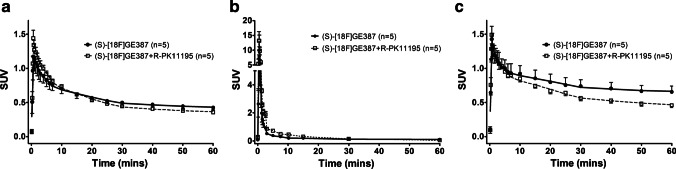
**a** Brain, **b** metabolite-corrected plasma and **c** olfactory bulb time–activity curves of (*S*)-[^18^F]GE387 with and without pre-treatment with 1 mg/kg of (*R*)-PK11195 in healthy naïve rats. Blocking effects of (*R*)-PK11195 are not observed to a significant extent in the healthy brain time–activity curves while plasma exposure is increased, possibly due to peripheral displacement of the radioligand

We next performed a blocking study in which rats were pre-treated with (*R*)-PK11195 (1 mg/kg) and found the (*S*)-[^18^F]GE387 whole-brain TAC SUV peak increased from 1.17 in naïve rats to 1.44 in rats pre-treated with (*R*)-PK11195 (Fig. 2). This was followed by a faster, but not statistically significant, washout (AUC = 32.1 ± 1.2) than observed with (*S*)-[^18^F]GE387 alone. Similar faster, but non-significant difference (AUC = 46.74 ± 6.1 in control vs 38.78 ± 1.7 in the blocked) in washout was observed in the olfactory bulb of animals pre-treated with (*R*)-PK11195. Plasma exposure of (*S*)-[^18^F]GE387 was statistically higher (*P* = 0.0223) after treatment with (*R*)-PK11195 (AUC = 25.23 ± 2.0, *n* = 5).

Biodistribution data from both enantiomers of [^18^F]GE387 are presented in Table 2. In the brain, 60 min after the radioligand injection, SUV was significantly higher in the olfactory bulb for (*S*)-[^18^F]GE387 (0.79 ± 0.06) compared to (*R*)-[^18^F]GE387 (0.43 ± 0.04). The uptake in the rest of the brain was low for both radioligands as expected in healthy naive rats. The uptake of (*S*)-[^18^F]GE387 in the olfactory bulb was significantly blocked by 49% by pre-treatment with (*R*)-PK11195. The uptake of (*S*)-[^18^F]GE387 in TSPO-rich peripheral organs such as the lungs, heart and adrenal glands was also blocked to a significant extent by (*R*)-PK11195 pre-treatment.

**Table 2:** Biodistribution data (SUV) at 60 min post-injection of (*R*)-[^18^F]GE387, (*S*)-[^18^F]GE387 and (*S*)-[^18^F]GE387 after pre-treatment with 1 mg/kg (*R*)-PK11195 (mean ± SEM). Differences between groups were examined separately for brain regions and peripheral tissues using 2-way ANOVA followed by post hoc Bonferroni test

Tissue	*(R)*-[^18^F]GE387 (*n* = 6)	*(S)*-[^18^F]GE387 (*n* = 9)	*P* value	*(S)*-[^18^F]GE387 + *(R)*-PK11195 (*n* = 5)	*P* value	% Reduction
Brain						
Olfactory bulb	0.43 ± 0.04	0.79 ± 0.06	< 0.001	0.40 ± 0.05	< 0.001	49
Striatum	0.27 ± 0.02	0.29 ± 0.03	ns	0.28 ± 0.04	ns	3
Cerebellum	0.32 ± 0.03	0.40 ± 0.03	ns	0.30 ± 0.03	ns	25
Pons and medulla	0.35 ± 0.05	0.43 ± 0.02	ns	0.34 ± 0.02	ns	22
Rest of the brain	0.28 ± 0.02	0.27 ± 0.02	ns	0.26 ± 0.02	ns	3
Periphery						
Pituitary gland	0.65 ± 0.12	1.86 ± 0.55	ns	0.66 ± 0.07	ns	64
Submandibular gland	1.25 ± 0.05	3.67 ± 0.22	ns	1.56 ± 0.35	ns	58
Thymus	1.03 ± 0.07	2.94 ± 0.27	ns	1.26 ± 0.26	ns	57
Lung	1.08 ± 0.07	9.64 ± 4.24	< 0.001	1.37 ± 0.29	< 0.001	86
Heart	1.07 ± 0.16	9.08 ± 0.63	< 0.001	1.53 ± 0.33	< 0.01	83
Liver	2.55 ± 0.18	2.17 ± 0.14		2.21 ± 0.20	ns	− 2
Spleen	0.92 ± 0.19	6.54 ± 0.49	< 0.05	1.53 ± 0.37	ns	77
Kidney	1.35 ± 0.12	6.90 ± 0.48	< 0.05	1.39 ± 0.22	ns	80
Adrenal gland	8.83 ± 0.60	43.21 ± 5.41	< 0.001	15.07 ± 4.92	< 0.001	65
Small intestine	2.93 ± 0.36	5.66 ± 0.58	ns	4.06 ± 1.09	ns	28
Large intestine	0.70 ± 0.05	2.57 ± 0.12	ns	0.88 ± 0.14	ns	66
Muscle	0.40 ± 0.03	0.50 ± 0.04	ns	0.39 ± 0.03	ns	22
Bone marrow	1.30 ± 0.15	4.51 ± 0.47	ns	2.27 ± 0.61	ns	50
Bone	0.55 ± 0.04	1.00 ± 0.14	ns	0.78 ± 0.15	ns	22
Urine	2.72 ± 0.79	0.33 ± 0.10	ns	0.49 ± 0.12	ns	− 47
Whole blood	0.47 ± 0.03	0.58 ± 0.24	ns	0.42 ± 0.04	ns	28
Plasma	0.67 ± 0.09	0.33 ± 0.04	ns	0.57 ± 0.07	ns	− 72
RBC	0.38 ± 0.09	0.19 ± 0.03	ns	0.32 ± 0.04	ns	− 67
Blood vessel	2.12 ± 0.45	3.81 ± 0.62	ns	0.91 ± 0.36	ns	76
Fat	0.87 ± 0.41	0.47 ± 0.07	ns	0.56 ± 0.17	ns	− 20

Kinetic modelling parameters from 2-TCM (Table 3) indicate that the blocking of TSPO with (*R*)-PK11195 significantly (*P* < 0.001) reduced the *V*_T_ of (*S*)-[^18^F]GE387 in the whole brain (3.52 ± 0.27 vs 1.90 ± 0.20); however individual rate constants, which are difficult to estimate precisely, did not show significant differences. In olfactory bulb, *V*_T_ (5.39 ± 0.34 vs 2.63 ± 0.38, *P* < 0.001) as well as BP_ND_ (3.39 ± 0.40 vs 2.36 ± 0.32, *P* < 0.01) was significantly reduced by (*R*)-PK11195 pre-treatment.

**Table 3:** 2-TCM rate constants and macro parameters (mean ± SEM) for (*S*)-[^18^F]GE387 obtained from the whole brain and olfactory bulb in the control animals and from animals pre-treated with (*R*)-PK11195. Differences between groups were examined separately for the whole brain and olfactory bulb using 2-way ANOVA followed by Bonferroni post hoc analysis for each of the parameters

Whole brain			
**2-TCM parameters**	**(*S*)-[**^**18**^**F]GE387 control (*n* = 9)**	**(*S*)-[**^**18**^**F]GE387 blocked (*n* = 5)**	***P* value**
*K*_1_	0.21 ± 0.02	0.14 ± 0.05	ns
*k*_2_	0.20 ± 0.01	0.19 ± 0.02	ns
*k*_3_	0.06 ± 0.004	0.04 ± 0.004	ns
*k*_4_	0.03 ± 0.002	0.02 ± 0.001	ns
*V*_T_	3.52 ± 0.27	1.90 ± 0.20	< 0.001
*K*_1_/*k*_2_	1.06 ± 0.05	0.71 ± 0.12	ns
*k*_3_/*k*_4_	2.30 ± 0.17	1.81 ± 0.25	ns
**Olfactory bulb**			
**2-TCM parameters**	**(*S*)-[**^**18**^**F]GE387 control (*n* = 8)**	**(*S*)-[**^**18**^**F]GE387 blocked (*n* = 5)**	***P* value**
*K*_1_	0.26 ± 0.02	0.15 ± 0.04	ns
*k*_2_	0.21 ± 0.03	0.19 ± 0.04	ns
*k*_3_	0.09 ± 0.02	0.06 ± 0.02	ns
*k*_4_	0.03 ± 0.002	0.02 ± 0.004	ns
*V*_T_	5.39 ± 0.34	2.63 ± 0.38	< 0.001
*K*_1_/*k*_2_	1.30 ± 0.14	0.83 ± 0.17	ns
*k*_3_/*k*_4_	3.39 ± 0.40	2.36 ± 0.32	< 0.01

### LPS neuroinflammation model

**Fig. 3:**
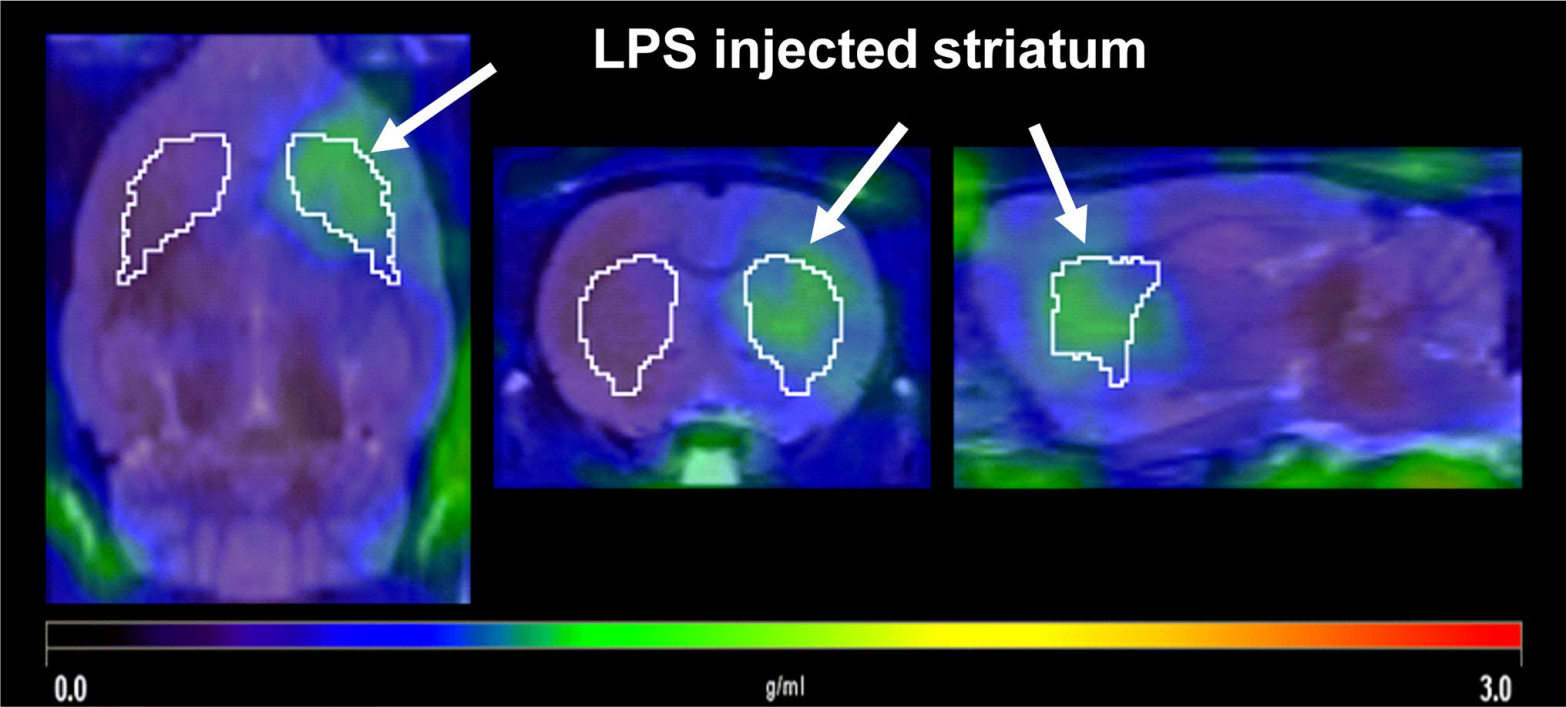
Summed SUV image of (*S*)-[^18^F]GE387 PET scan (30–60 min) in an LPS-injected rat overlaid on an MRI template. Higher uptake of (*S*)-[^18^F]GE387 in the LPS-injected striatum (arrow) compared to the saline-injected contralateral striatum shown in transverse, coronal and sagittal views

**Fig. 4:**
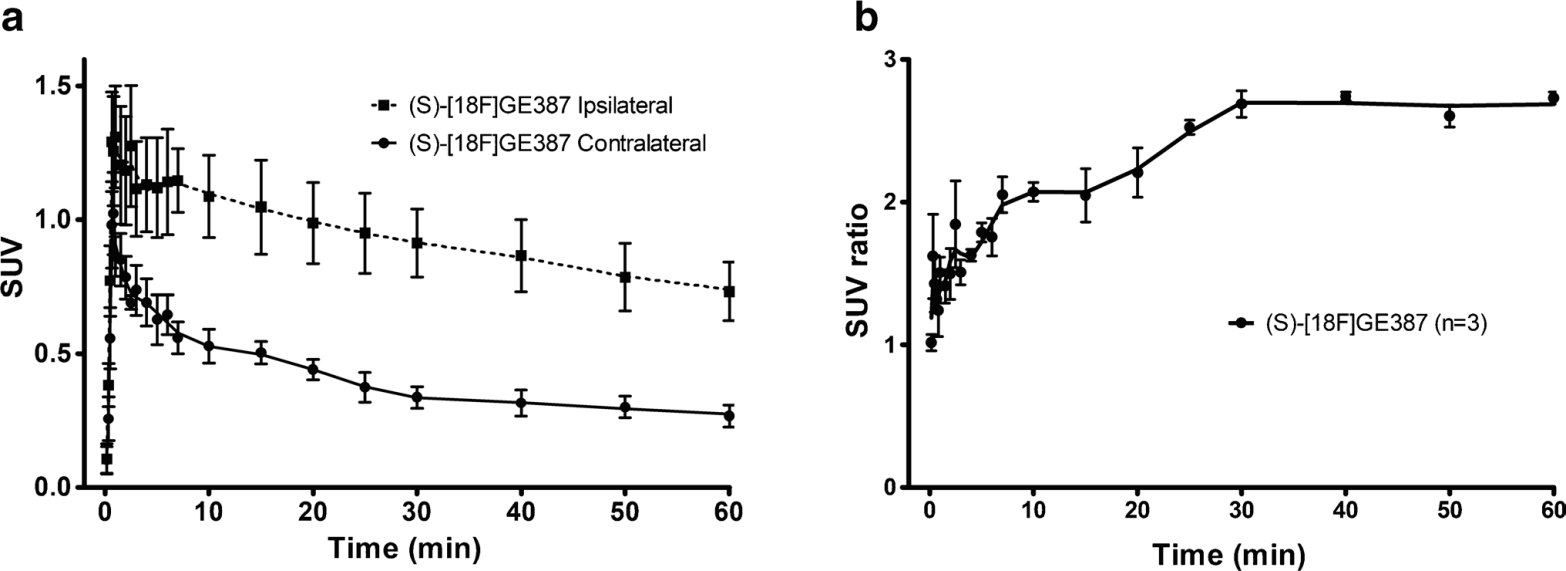
**a** Time–activity curves of (*S*)-[^18^F]GE387 in ipsilateral and contralateral striatum 3 days after LPS injection. **b** Ipsilateral to contralateral SUV ratio stabilised at around 2.7 from 30 min post-injection

In the PET scan images of the LPS-injected rats, higher uptake of (*S*)-[^18^F]GE387 was seen on the ipsilateral striatum (Fig. 3) compared to the saline-injected contralateral striatum. TACs showed rapid uptake of (*S*)-[^18^F]GE387 in both ipsilateral and contralateral striata. While the tracer was fairly rapidly washed out from the contralateral striatum (AUC = 24.3 ± 2.9, *n* = 3), the washout was much slower from the ipsilateral striatum (Fig. 4a) and the SUV remained higher than the contralateral striatum throughout the 60-min scan (AUC = 55.7 ± 8.5, *n* = 3). The ipsilateral to contralateral SUV ratio stabilised from 30 min (Fig. 4b), and an SUV (30–60 min) ratio of 2.7 was obtained which compares favourably with the SUV ratio of 2.4 obtained from [^18^F]DPA714 scans (supplementary information).

2-TCM modelling parameters (Table 4) for (*S*)-[^18^F]GE387 from the noninflamed contralateral striatum did not differ significantly from that of the striatum of naïve healthy controls. Significant differences (*P* < 0.001) were observed in the *V*_T_ and BP_ND_ between the ipsilateral and contralateral striatum. While there is a trend towards higher *K*_1_ and *k*_2_ values for the inflamed striatum, it did not reach significance. The ipsilateral to contralateral BP_ND_ ratio (2.97) for (*S*)-[^18^F]GE387 was not significantly different from that of [^18^F]DPA714 (2.15) (supplementary information).

**Table 4:** 2-TCM parameters for (*S*)-[^18^F]GE387 obtained from the striata in the control animals and from contralateral and ipsilateral striata in the LPS neuroinflammation model (mean ± SEM). Differences between groups were examined using 2-way ANOVA followed by Bonferroni post hoc analysis for each of the parameters

2-TCM parameters	(*S*)-[^18^F]GE387 control (*n* = 9)	(*S*)-[^18^F]GE387 contralateral (*n* = 3)	*P*	(*S*)-[^18^F]GE387 ipsilateral (*n* = 3)	*P*
*K*_1_	0.19 ± 0.02	0.25 ± 0.09	ns	0.52 ± 0.19	ns
*k*_2_	0.24 ± 0.04	0.30 ± 0.11	ns	0.71 ± 0.33	ns
*k*_3_	0.07 ± 0.03	0.12 ± 0.05	ns	0.35 ± 0.14	ns
*k*_4_	0.03 ± 0.01	0.06 ± 0.02	ns	0.07 ± 0.01	ns
*V*_T_	2.40 ± 0.22	2.35 ± 0.41	ns	5.28 ± 0.92	< 0.001
*K*_1_/*k*_2_	0.89 ± 0.09	0.95 ± 0.16	ns	1.12 ± 0.41	ns
BP_ND_ = *k*_3_/*k*_4_	1.86 ± 0.28	1.60 ± 0.49	ns	5.10 ± 1.91	< 0.001

### Non-human primates

**Fig. 5:**
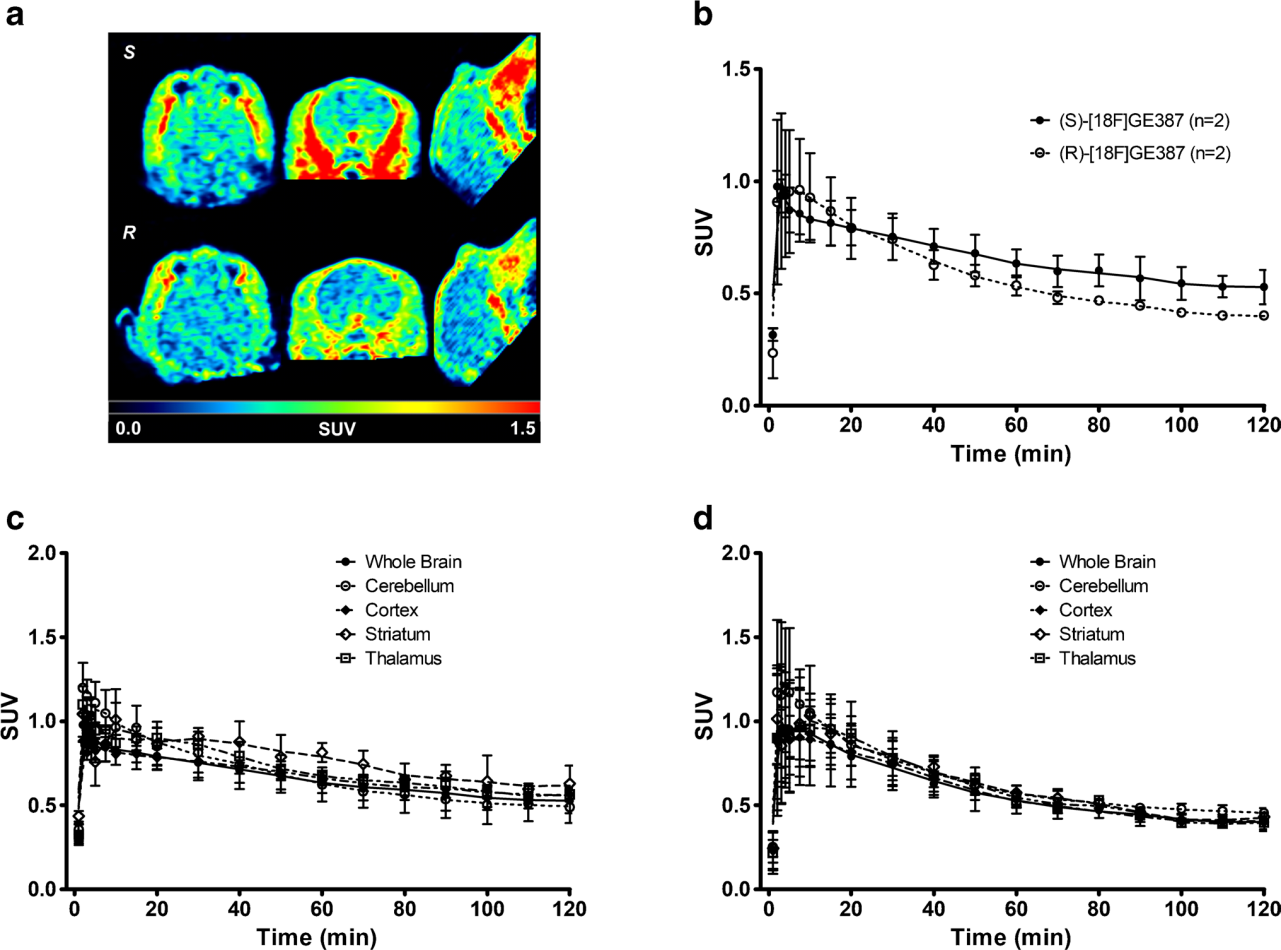
**a** Summed (*S*)-[^18^F]GE387 and (*R*)-[^18^F]GE387 SUV image (60–120 min) of the brain in the same rhesus macaque. **b** Time–activity curves of (*S*)-[^18^F]GE387 and (*R*)-[^18^F]GE387 in the whole brain in two rhesus macaques. Both radioligands entered the brain with a peak within the first 2 min while (*R*)-[^18^F]GE387 had a faster washout compared to (*S*)-[^18^F]GE387. **c** Average (*n* = 2) (*S*)-[^18^F]GE387 time–activity curves in the whole brain, cerebellum, cortex, striatum and thalamus. **d** Average (*n* = 2) (*R*)-[^18^F]GE387 time–activity curves in the whole brain, cerebellum, cortex, striatum and thalamus. (*R*)-[^18^F]GE387 had a more uniform distribution of uptake within the regions of the brain compared to (*S*)-[^18^F]GE387

**Fig. 6:**
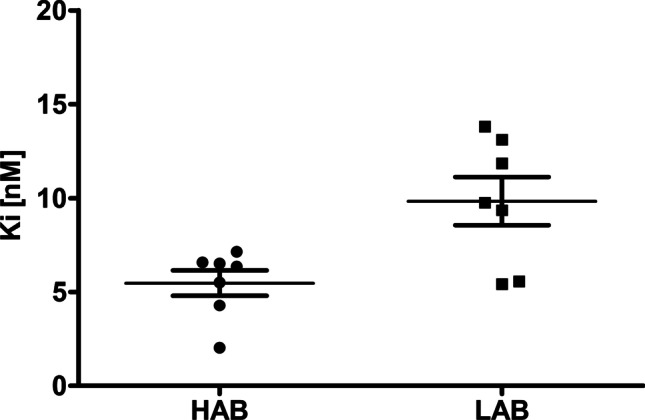
*K*_*i*_ values obtained from a [^3^H]PK11195 competition binding assay of unlabelled (*S*)-GE387. While the difference in *K*_*i*_ between the two groups was statistically significant by unpaired *t*-test (*P* = 0.0108), the calculated LAB to HAB binding ratio was low at 1.8

Both (*S*)-[^18^F]GE387 and (*R*)-[^18^F]GE387 were found to enter the brain in the two rhesus macaques (Fig. 5). The time–activity curves of (*S*)-[^18^F]GE387 and (*R*)-[^18^F]GE387 showed a similar trend as seen in the Wistar rats (Fig. 6). Maximal brain uptake was observed within 2 min of injection for both tracers followed by a more rapid washout for the *R*-enantiomer (AUC = 68) than the *S*-enantiomer (AUC = 81).

### Competition binding assay

A competition binding assay using unlabelled (*S*)-GE387 against [^3^H]PK11195 yielded *K*_*i*_ of 5.48 ± 0.68 nM (*n* = 7) for HABs and 9.83 ± 1.28 nM (*n* = 7) for LABs (Fig. 6). While the difference in *K*_*i*_ between the two groups was statistically significant by unpaired *t*-test, (*P* = 0.0108), the calculated LAB to HAB binding ratio was low (1.8:1).

## Discussion

In this paper, we have evaluated the suitability of (*S*)-[^18^F]GE387 and (*R*)-[^18^F]GE387 for use as radioligands for imaging neuroinflammation. (*S*)-[^18^F]GE387 and (*R*)-[^18^F]GE387 behaved similarly in rats and rhesus macaques with a rapid uptake of the radioligands in the brain followed by a slow washout. Consistent with the low expression of TSPO in normal brain tissue [20], and as seen with other TSPO PET radioligands [21], in healthy rats and rhesus macaques, the brain uptake of both radioligands was found to be low.

While blood sampling was not permitted in the macaques, blood, brain and other tissue sampling in rats allowed the evaluation of the formation of radioactive metabolites. (*S*)-[^18^F]GE387 had a better metabolite profile than (*R*)-[^18^F]GE387 in the plasma, which at 28% at 60 min post-injection is similar to [^18^F]GE180 (21 ± 4.6%) [22] and [^18^F]PBR111 (20%) [23]. (*S*)-[^18^F]GE387 had slower metabolism than (*R*)-[^18^F]GE387 and also had lower levels of radiometabolites in the brain at the end of the 60-min scan. While 78% parent in the rat brain is comparatively low, we believe the single solvent extraction procedure in the lipid-rich brain could skew the extraction towards the more polar metabolite over the less polar parent.

In the biodistribution study, the uptake of (*S*)-[^18^F]GE387 was higher than that of (*R*)-[^18^F]GE387 in TSPO-rich organs of the periphery and was blocked by pre-treatment with (*R*)-PK11195 (Table 2). The only region of the healthy brain where the already low uptake of (*S*)-[^18^F]GE387 was blocked by (*R*)-PK11195 and therefore found to be specific uptake was in the olfactory bulb where TSPO is constitutively expressed in normal rat brain [20, 24]. The other brain regions with low constitutive expression of TSPO showed non-significant effects of the blocking, which could also be due to the higher plasma exposure created by blocking of peripheral TSPO. The low percentage reduction of 3% observed in the striatum makes this region a suitable region for induction of neuroinflammation with low background uptake. Kinetic modelling corroborated this blocking effect in the olfactory bulb where the binding potential was significantly reduced compared with the whole brain where only the *V*_T_ was significantly reduced.

Based on its better biological profile, (*S*)-[^18^F]GE387 was tested in a rat model of neuroinflammation where injection of LPS into one striatum induces an inflammatory response and the vehicle-injected contralateral striatum serves as an internal control. The inflamed striatum was reliably differentiated from its internal control region by (*S*)-[^18^F]GE387 PET imaging with an uptake 2.7 times that of the control side (Fig. 4). This value is similar to that obtained for [^18^F]DPA714 in our laboratory (Supplementary Fig. 2) as well as for (*R*)-[^11^C]PK11195, [^18^F]GE-180 and [^18^F]DPA714 in similar models [21]. Having a comparable ratio between inflamed and normal tissue to [^18^F]DPA714 while having a lower background uptake could give (*S*)-[^18^F]GE387 a better signal-to-noise ratio. Binding potential ratio (inflamed to normal striatum) of (*S*)-[^18^F]GE387 (2.97) was also comparable to that obtained with [^18^F]DPA714 (2.15) (Supplementary table 3).

We have previously shown that (*S*)-[^18^F]GE387 has a LAB to HAB binding ratio of 1.3 in a cell-based assay using human embryonic kidney cell lines stably overexpressing wild-type human TSPO and A147T TSPO [15]. However, results from such cell-based assays sometimes differ from those obtained using human tissue, for example, GE180 gave a value of 5.1:1 [15] in the same cell-based assay, while a ratio of 15:1 [11, 12] was reported in a human brain tissue assay. Therefore, we evaluated (*S*)-GE387 in a human brain tissue competition binding assay against [^3^H]PK11195. In addition to (*S*)-GE387 yielding low nanomolar *K*_*i*_ values in both HABs and LABs, the LAB to HAB ratio of 1.8:1 obtained for (*S*)-GE387 is one of the lowest (see Table 2) of all the later TSPO radioligands [9]. [^11^C]ER176, which has the lowest LAB to HAB ratio of the clinically tested TSPO radiotracer, is hindered by being radiolabelled with the short-lived radioisotope carbon-11 and the in vitro prediction of low sensitivity to polymorphism was not replicated in human in vivo studies [25]. Recently, novel TSPO radioligands that are at the preclinical stages of evaluation have been reported with low LAB to HAB binding ratio in human thrombocyte membranes ((*R*)-[^18^F]NEBIFQUINIDE, LAB to HAB ratio 1.1 [13]) and in human brain tissue ([^18^F]LW223, LAB to HAB ratio 1 [14]). Both radioligands appear to have good in vitro and preclinical in vivo properties. (*R*)-[^18^F]NEBIFQUINIDE, however, has not yet been tested in a model of neuroinflammation while [^18^F]LW223 has been tested in a model of myocardial infarction–induced inflammation in the heart where brain inflammation axis was investigated.

While the development of alternatives to TSPO imaging for assessing neuroinflammation is an active field, as of yet, none of the new biomarkers and/or their selective radioligands has been shown to be able to successfully assess neuroinflammatory pathology in humans [26, 27]. Given the vast body of knowledge derived from TSPO PET [5], it remains the “gold standard” approach for quantification of neuroinflammation in humans. Therefore, based on the favourable properties of (*S*)-[^18^F]GE387 identified herein and the need to confirm the observations in human studies, further evaluation in human subjects is warranted.

## Conclusion

In this study, we have established that (*S*)-[^18^F]GE387 has low background uptake and favourable kinetics in healthy rats and non-human primates. Its specific uptake in the brain and TSPO-rich peripheral organs could be blocked by pre-treatment with established TSPO ligand (*R*)-PK11195. Crucially, PET imaging with (*S*)-[^18^F]GE387 could be used to differentiate between inflamed and normal brain tissue in a rat model of neuroinflammation, which compared favourably with a leading TSPO radioligand. We have additionally confirmed its low sensitivity to TSPO polymorphism in genotyped human brain tissue. Further studies, such as metabolite identification, dosimetry and toxicology, are required, to support the translation of this radioligand to the clinic and hence determine if (*S*)-[^18^F]GE387 has potential for imaging neuroinflammation in human subjects irrespective of genotype.

## Supplementary Information

Below is the link to the electronic supplementary material.

Supplementary file1 (DOCX 3494 KB)

## Data Availability

The datasets used and/or analysed during the current study are available from the corresponding author on reasonable request.

## Abbreviations

TSPO: 18-KDa translocator protein
PET: Positron emission tomography
LPS: Lipopolysaccharide
LAB: Low affinity binder
HAB: High affinity binder
MAB: Mixed affinity binder
HPLC: High-performance liquid chromatography
VOI: Volume of interest
SUV: Standardised uptake value
MRI: Magnetic resonance imaging
TAC: Time–activity curve
AUC: Area under the curve
