# Supplementary material for: Preclinical evaluation of (S)-[18F]GE387, a novel 18-kDa translocator protein (TSPO) PET radioligand with low binding sensitivity to human polymorphism rs6971
Source: Eur J Nucl Med Mol Imaging. 2021 Aug 18;49(1):125–36. doi: 10.1007/s00259-021-05495-w (PMC8712295; doi:10.1007/s00259-021-05495-w)
Supplement: Supplementary file 1 — Supplementary file1 (DOCX 3494 KB) [file 259_2021_5495_MOESM1_ESM.docx]

**Supplementary Data:**

**Preclinical evaluation of *(S)*-[^18^F]GE387, a novel 18 kDa translocator protein (TSPO) PET radioligand with low binding sensitivity to human polymorphism rs6971**

**Authors**

Nisha K. Ramakrishnan^1*^, Matthew Hird^1^, Stephen Thompson^1^, David J. Williamson^1^, Luxi Qiao^1^, David R. Owen^2^, Allen F. Brooks^3^, Peter J.H. Scott^3^, Sergio Bacallado^4^, John O'Brien^5^ and Franklin I. Aigbirhio^1^

**Affiliations**

1. Molecular Imaging Chemical Laboratory, Wolfson Brain Imaging Centre, Department of Clinical Neurosciences, University of Cambridge, Biomedical Campus, Cambridge CB2 0SZ (UK),

2. Division of Experimental Medicine, Department of Medicine, Imperial College London, Hammersmith Hospital, London (UK),

3. Division of Nuclear Medicine, Department of Radiology, University of Michigan Medical School, 1301 Catherine Street, Ann Arbor, Michigan 48109, United States

4. Statistical Laboratory, Centre for the Mathematical Sciences, Wilberforce Rd. Cambridge, CB3 0WB (UK)

5. Department of Psychiatry, School of Clinical Medicine, University of Cambridge, Cambridge Biomedical Campus, Cambridge, UK

* Correspondence: nk473@cam.ac.uk

Synthesis of (*S*)-[^18^F]GE387 and (*R*)-[^18^F]GE387

(*S*)-[^18^F]GE387 and (*R*)-[^18^F]GE387 were synthesised from their corresponding enantiomerically pure precursors using the method previously reported (see supplementary information) (1). Briefly, no-carrier-added [^18^F]fluoride was produced *via* the ^18^O(p,n)^18^F nuclear reaction from enriched [^18^O]water using a GE PETtrace cyclotron and was trapped on a preconditioned Sep-Pak Carbonate QMA cartridge. The trapped [^18^F]fluoride was eluted from the cartridge with kryptofix-222 and K_2_CO_3_ (0.25% *w/v* and 0.05% (*w/v*) respectively) in aqueous MeCN (75% MeCN *v/v,* 1 mL) into the reactor of a GE Healthcare F_X_F_N_ TRACERlab. The solvents were evaporated in vacuo (130 mbar) with a stream of N_2_ gas at 95 °C over 5 min. Anhydrous MeCN (3×0.7 mL) was then added and the mixture was azeotropically dried in vacuo (130 mbar) with a stream of N_2_ at 95 °C. The tosyl precursor for either (*S*)-[^18^F]GE387 or (*R*)-[^18^F]GE387 (1-1.5 mg) in anhydrous MeCN (1 mL) was added, and the reactor heated to 100 °C for 20 minutes. The crude reaction mixture was diluted into HPLC buffer (2 mL), and purified by semi-preparative reverse-phase HPLC (ACE 160433, C_18_, 5 μm, 100×10 mm, 48 % aq. MeCN at a flow rate of 5 mL min^−1^). Unreacted [^18^F]fluoride eluted at 2 minutes and the radiolabelled [^18^F]GE387 product was collected between 17-23 minutes, and diluted into H_2_O (30 mL). The aqueous solution was passed through a C_18_ cartridge (Waters Sep-Pak Accell Light C_18_ cartridge). The cartridge was washed with H_2_O (10 mL) and the radiolabelled product was eluted with EtOH (0.5 mL), followed by formulation with saline (0.9%, 4.5 mL) to provide the radiolabelled product as a solution in 10% *v/v* EtOH in 0.9% saline.

From end-of-bombardment (EOB), the total time of the automated radiosynthesis was 60 min. Radiochemical purity was >98%, and non-decay corrected radiochemical yields and molar activities for (*R*)-[^18^F]GE387 and (*S*)-[^18^F]GE387 were 21.3 ± 16.7% and 25.6 ± 7.1%, and 55.8 ± 35.6 GBq.µmol^-1^ and 63.5 ± 39.5 GBq.µmol^-1^ (n = 3) respectively at the end-of-synthesis.

Synthesis of [^18^F]DPA-714

[^18^F]DPA714 was prepared similar to published method(2). No-carrier-added [^18^F]fluoride was produced via nuclear ^18^O(p, n)^18^F reaction from enriched ^18^O-water using GE PETtrace cyclotron and it was immediately trapped on a preconditioned Sep-Pak Carbonate QMA cartridge. The trapped [^18^F]fluoride was eluted from the cartridge with 0.25% wt Kryptofix222 solution (1 mL) in basic (0.05% wt K2CO3) aq. MeCN (75% vv) into a tightly closed reactor in the GE Healthcare FX_FN_ TRACERlab instrument. The solvents were evaporated in vacuo (130 mbar) with a stream of N_2_ gas at 95 °C over 5 min. Anhydrous MeCN (3×0.7 mL) was then added and the mixture was azeotropically dried in vacuo (130 mbar) with a stream of N_2_ at 95 °C. Then 4-5 mg of the tosyl precursor in dimethyl sulfoxide was added to the reactor, which was heated to 165°C for 5 min. After cooling, the reaction mixture was diluted and passed through a Sep-Pak® Alumina N cartridge to eliminate free [^18^F]Fluoride. Crude material was then purified using semi-preparative HPLC with a retention time of 9 min. The product was then formulated in 10% ethanol in saline before eluting from the module. From EOB, the time of the automated radiosynthesis is 40 min. Radiochemical purity was >98%, and the radiolabelling yield was 10.3 ± 0.3% decay corrected to EOB or 7.8 ± 0.5% non-decay corrected. The molar activity was 108.6 ± 30.1 GBq/μmol at the EOS (n = 3).

**Supplementary Table 1:** Details of the number of rats in each group, their weights and injected doses

| **Radioligand** | **Number of animals** | **Weight (g)** | | | **Injected dose (MBq)** | | |
| --- | --- | --- | --- | --- | --- | --- | --- |
| (*S*)-[^18^F]GE387 |  | | | | | | |
| AIF sampling | 5 | 360 | ± | 37 | 36 | ± | 8 |
| Metabolite sampling only | 4 | 472 | ± | 21 | 81 | ± | 5 |
| (*R*)-[^18^F]GE387 |  | | | | | | |
| AIF sampling | 5 | 339 | ± | 25 | 40 | ± | 10 |
| Metabolite sampling only | 3 | 507 | ± | 38 | 91 | ± | 8 |
| (*S*)-[^18^F]GE387 + (*R*)-PK11195 | 5 | 382 | ± | 43 | 37 | ± | 5 |
| (*S*)-[^18^F]GE387 in LPS model | 3 | 341 | ± | 14 | 33 | ± | 1 |
| [^18^F]DPA714 | 3 | 372 | ± | 11 | 59 | ± | 13 |
| [^18^F]DPA714 in LPS model | 3 | 416 | ± | 94 | 24 | ± | 6 |

[^18^F]DPA-714 TAC in healthy rats





**Supplementary Figure 1:** [^18^F]DPA714 PET scans were performed in naïve healthy rats as for (*S*)-[^18^F]GE387. Maximal brain uptake was within one minute for [^18^F]DPA714 with a peak SUV of 1.1 which was followed by a slower washout from the rat brain though the AUC (42.4 ± 2.97) was not statistically higher (*P* = 0.0968) than that for (*S*)-[^18^F]GE387 (AUC=33.6 ± 2.9).

[^18^F]DPA-714 TAC in LPS neuroinflammation model

**

**

**Supplementary Figure 2:** [^18^F]DPA714 PET scans in the LPS neuroinflammation model were performed as for (*S*)-[^18^F]GE387. A) While the tracer was fairly rapidly washed out from the contralateral striatum (AUC = 45.46 ± 3.201 N=3), the wash out was much slower from the ipsilateral striatum and the SUV remained higher than the contralateral striatum throughout the 60 minute scan (AUC = 95.94 ± 6.224 N=3). B). The ipsilateral to contralateral SUV ratio stabilised from 30 minutes and an SUV (30 – 60 min) ratio of 2.4 was obtained.

Kinetic Modelling of (*S*)-[^18^F]GE387

Kinetic modelling of (*S*)-[^18^F]GE387 data was performed using PMOD software (version 3.8; PMOD technologies, Zurich, Switzerland). Blood and plasma time-activity curves (TACs) were interpolated by fitting a 3-exponentials model. Where whole blood or plasma curves were not available (due to metabolite sampling protocol or failure of cannula), population averages for the group corrected for injected dose and weight of individual animal was used. Population average metabolite curve fractions were used for correcting plasma curves. The parameter for cerebral Blood volume (vB) was fixed at 3.6% and blood delay was fitted.

1-tissue compartment model (1-TCM), 2-tissue compartment model (2-TCM), Logan graphical analysis and Simplified Reference Tissue Model (SRTM) were fitted to brain TACs from the (*S*)-[^18^F]GE387 control animals, those pretreated with (*R*)-PK1195 and the LPS treated animals.

2-TCM, was found to be better compared to 1-TCM based on fit and Akaike information criterion (Supplementary figure 3).





**Supplementary Figure 3:** Akaike Information Criterion for the various models

To simplify future analysis, Logan graphical analysis was compared against 2-TCM. The fit was started at 15 min, vB was fixed at 3.6 % and blood delay fitted.

For 2-TCM of smaller brain regions, K_1_/k_2_ estimated from individual whole brain was fixed for estimation of total volume of distribution (V_T_) and non-displaceable binding Potential (BP_ND_).





**Supplementary Figure 4:** V_T_ of brain regions from 2-TCM was compared against Logan graphical analysis. V_T_ from Logan graphical analysis correlated strongly with V_T_ from 2-TCM (r^2^ = 0.9376, *P* < 0.0001) and was found to underestimate V_T_ by about 3%.

Kinetic modelling of data from LPS neuroinflammation model

**Supplementary Table 3:** 2-TCM parameters obtained from the striata in the (*S*)-[^18^F]GE387 control animals and from (*S*)-[^18^F]GE387 and [^18^F]DPA714 LPS neuroinflammation model (Mean ± SEM). Sufficient arterial input functions were not available in the [^18^F]DPA714 control group to perform 2-TCM analysis

| **2-TCM parameters** | **(*S*)-[^18^F]GE387 Control (n=9)** | | | **(*S*)-[^18^F]GE387 Contralateral (n=3)** | | | **(*S*)-[^18^F]GE387 Ipsilateral (n=3)** | | | **[^18^F]DPA714 Contralateral (n=3)** | | | **[^18^F]DPA714 Ipsilateral (n=3)** | | |
| --- | --- | --- | --- | --- | --- | --- | --- | --- | --- | --- | --- | --- | --- | --- | --- |
| K1 | 0.19 | ± | 0.02 | 0.25 | ± | 0.09 | 0.52 | ± | 0.19 | 1.05 | ± | 0.08 | 1.75 | ± | 0.09 |
| k2 | 0.24 | ± | 0.04 | 0.30 | ± | 0.11 | 0.71 | ± | 0.33 | 0.17 | ± | 0.02 | 0.19 | ± | 0.04 |
| k3 | 0.07 | ± | 0.03 | 0.12 | ± | 0.05 | 0.35 | ± | 0.14 | 0.06 | ± | 0.01 | 0.13 | ± | 0.06 |
| k4 | 0.03 | ± | 0.01 | 0.06 | ± | 0.02 | 0.07 | ± | 0.01 | 0.03 | ± | 0.01 | 0.03 | ± | 0.01 |
| Vs (=K1/k2*k3/k4) | 1.51 | ± | 0.17 | 1.41 | ± | 0.42 | 4.16 | ± | 1.06 | 12.24 | ± | 1.48 | 37.25 | ± | 2.35 |
| Vt | 2.40 | ± | 0.22 | 2.35 | ± | 0.41 | 5.28 | ± | 0.92 | 18.40 | ± | 1.67 | 48.07 | ± | 1.76 |
| K1/k2 | 0.89 | ± | 0.09 | 0.95 | ± | 0.16 | 1.12 | ± | 0.41 | 6.15 | ± | 0.23 | 10.82 | ± | 2.91 |
| k3/k4 | 1.86 | ± | 0.28 | 1.60 | ± | 0.49 | 5.10 | ± | 1.91 | 1.98 | ± | 0.19 | 4.19 | ± | 1.48 |
| BP_ND_ ratio |  |  |  | 2.97 ± 0.54 | | | | | | 2.15 ± 0.80 | | | | | |

Comparing the BP_ND_ (ipsilateral to contralateral) ratios of (*S*)-[^18^F]GE387 and [^18^F]DPA714 gives values of 2.97 and 2.15 respectively. They are not significantly different . This is comparable to the BP_ND_ ratio (3.0) that was obtained by Ory et al.(3) for [^18^F]DPA714 although Ory et al. applied 50ug of LPS to induce the neuroinflammation compared to 10ug that we applied.

References

1. Qiao L, Fisher E, McMurray L, Milicevic Sephton S, Hird M, Kuzhuppilly-Ramakrishnan N, et al. Radiosynthesis of (R,S)-[ ^18^ F]GE387: A Potential PET Radiotracer for Imaging Translocator Protein 18 kDa (TSPO) with Low Binding Sensitivity to the Human Gene Polymorphism rs6971. ChemMedChem. 2019;14(9).

2. James ML, Fulton RR, Vercoullie J, Henderson DJ, Garreau L, Chalon S, et al. DPA-714, a New Translocator Protein-Specific Ligand: Synthesis, Radiofluorination, and Pharmacologic Characterization. J Nucl Med [Internet]. 2008 Apr 15;49(5):814–22. Available from: http://jnm.snmjournals.org/cgi/doi/10.2967/jnumed.107.046151

3. Ory D, Postnov A, Koole M, Celen S, de Laat B, Verbruggen A, et al. Quantification of TSPO overexpression in a rat model of local neuroinflammation induced by intracerebral injection of LPS by the use of [18F]DPA-714 PET. Eur J Nucl Med Mol Imaging [Internet]. 2016 Jan 1;43(1):163–72. Available from: http://link.springer.com/10.1007/s00259-015-3172-9
